# Electronic Topological Transition as a Route to Improve Thermoelectric Performance in Bi_0.5_Sb_1.5_Te_3_

**DOI:** 10.1002/advs.202105709

**Published:** 2022-03-15

**Authors:** Feng‐Xian Bai, Hao Yu, Ya‐Kang Peng, Shan Li, Li Yin, Ge Huang, Liu‐Cheng Chen, Alexander F. Goncharov, Jie‐He Sui, Feng Cao, Jun Mao, Qian Zhang, Xiao‐Jia Chen

**Affiliations:** ^1^ School of Materials Science and Engineering and Institute of Materials Genome & Big Data Harbin Institute of Technology Shenzhen 518055 China; ^2^ Center for High Pressure Science and Technology Advanced Research Shanghai 201203 China; ^3^ School of Science Harbin Institute of Technology Shenzhen 518055 China; ^4^ Earth and Planets Laboratory Carnegie Institution for Science Washington D.C. 20015 USA; ^5^ State Key Laboratory of Advanced Welding and Joining Harbin Institute of Technology Harbin 150001 China

**Keywords:** Bi_0.5_Sb_1.5_Te_3_, electronic topological transition, pressure, thermoelectric materials

## Abstract

The electronic structure near the Fermi surface determines the electrical properties of the materials, which can be effectively tuned by external pressure. Bi_0.5_Sb_1.5_Te_3_ is a p‐type thermoelectric material which holds the record high figure of merit at room temperature. Here it is examined whether the figure of merit of this model system can be further enhanced through some external parameter. With the application of pressure, it is surprisingly found that the power factor of this material exhibits λ behavior with a high value of 4.8 mW m^−1^ K^−2^ at pressure of 1.8 GPa. Such an enhancement is found to be driven by pressure‐induced electronic topological transition, which is revealed by multiple techniques. Together with a low thermal conductivity of about 0.89 W m^−1^ K^−1^ at the same pressure, a figure of merit of 1.6 is achieved at room temperature. The results and findings highlight the electronic topological transition as a new route for improving the thermoelectric properties.

## Graphical abstract

This paper reports the pressure‐induced electronic topological transition as a novel route for improving thermoelectric performance. With the application of high pressure, the room‐temperature *zT* of Bi_0.5_Sb_1.5_Te_3_ exhibits a λ behavior, and the maximum *zT* value is as high as  1.6 around the vicinity of the electronic topological transition.

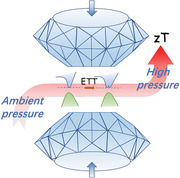

## 1 Introduction

Thermoelectric (TE) technology, which can convert heat energy into electricity and vice versa, has attracted extensive attention due to its potential to realize environmentally friendly and high‐efficiency energy conversion.^[^ ^1^, ^2^, ^3^ ^]^ The energy conversion efficiency of TE materials is governed by the dimensionless figure of merit *zT* = $\sigma S^{2}$ *T*/κ, where σ, *S*, κ, and *T* are the electrical conductivity, Seebeck coefficient, thermal conductivity (including the lattice thermal conductivity κ_L_ and electronic thermal conductivity κ_e_), and absolute temperature, respectively.^[^ ^4^, ^5^ ^]^ Excellent TE materials should have high σ and *S*, and low κ. The strong coupling between electrons and phonons retards the increase of *zTs*. In the past several decades, abundant works have been progressing to further improve the TE performance through nanostructuring,^[^ ^6^, ^7^, ^8^ ^]^ band engineering,^[^ ^9^, ^10^, ^11^ ^]^ optimizing carrier concentration,^[^ ^12^, ^13^, ^14^ ^]^ and defect engineering,^[^ ^15^, ^16^, ^17^ ^]^ etc. However, the significant improvements on the room‐temperature *zT* is still highly desired for the technological applications.

Bi_2_Te_3_‐based alloys are well‐known TE materials for solid‐state refrigeration and power generation near room temperature.^[^ ^18^, ^19^ ^]^ Varieties of strategies have been developed to improve their room‐temperature *zT*s.^[^ ^8^, ^20^, ^21^, ^22^, ^23^, ^24^, ^25^, ^26^, ^27^ ^]^ A high room‐temperature *zT* of ≈1.2 was obtained in nanostructured Bi_0.4_Sb_1.6_Te_3_ due to the intensive phonon scattering.^[^ ^8^ ^]^ By using a simple hot‐forging process on p‐type Bi_0.5_Sb_1.5_Te_3_ to induce in‐situ nanostructures and dense defects, an enhanced *zT* value of more than 1.3 at room temperature was obtained.^[^ ^26^ ^]^ Another remarkable enhanced room‐temperature *zT* value to 1.4 was realized by embedding superparamagnetic Fe_3_O_4_ nanoparticles in a p‐type Bi_0.5_Sb_1.5_Te_3_ alloy due to the carrier multiple scattering for a high *S* and magnetic moment fluctuation for a low lattice thermal conductivity.^[^ ^27^ ^]^

As a basic thermodynamic variable, pressure can change many physical properties that include crystal structure, electronic, and magnetic structure etc. without introducing impurities,^[^ ^28^ ^]^ which contributes to the acquirement of large *zTs* at certain pressures. Notably enhanced TE properties have been widely achieved by imposing pressure on many TE materials.^[^ ^29^, ^30^, ^31^, ^32^, ^33^, ^34^, ^35^ ^]^ Especially, the pressure‐driven topological phase transition in Pb_0.99_Cr_0.01_Se was reported to give rise to a high room‐temperature *zT* value of about 1.7 at the pressure of 3 GPa.^[^ ^31^ ^]^ A largely enhanced power factor by more than 100 over a wide temperature range (10–300 K) was obtained in SnSe, which was attributed to the pressure‐induced electronic topological transition (ETT).^[^ ^32^ ^]^ ETT is described by the collapse or emergence of Fermi surfaces that can be induced by external parameters, such as pressure or temperature. The occurring of ETT is always accompanied by anomalies in the density of states which is anticipated to strongly affect the transport phenomena. Since seebeck coefficient is proportional to the energy derivative of the density of states, the thermoelectric performance of materials can be thus further tuned via the introducing of ETT.^[^ ^33^ ^]^

In this work, we employ the external pressure to further improve the room‐temperature *zT* of Bi_0.5_Sb_1.5_Te_3_ in view of the existence of the pressure‐induced ETT in this alloy.^[^ ^36^, ^37^ ^]^ A high power factor of   4.8 mW m^−1^ K^−2^ with a low κ of   0.89 Wm^−1^ K^−1^ at 1.8 GPa is achieved at room temperature. As a result, *zT* is increased dramatically with pressure, reaching 1.6 at ≈1.8 GPa.

## 2 Results and Discussion

**Figure 1:**
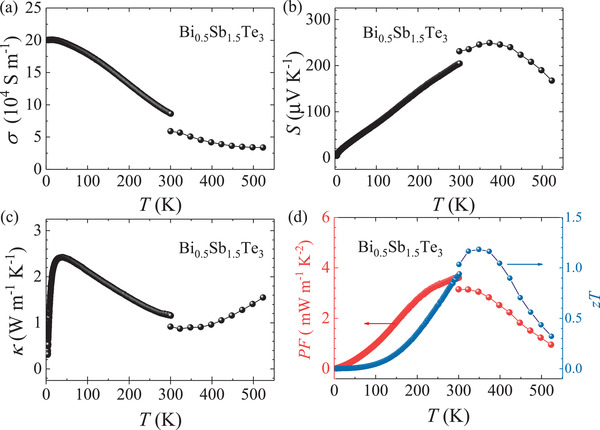
Temperature‐dependent a) electrical conductivity (σ), b) Seebeck coefficient *S*, c) thermal conductivity κ, d) PF (red, left panel) and zT value (blue, right panel) of Bi_0.5_Sb_1.5_Te_3_.

The temperature‐dependent TE properties of Bi_0.5_Sb_1.5_Te_3_ were measured from 2 to 525 K at ambient pressure and at temperatures at below 300 K by using physical properties measurement system (PPMS). A commercial system (CTA) was employed to measure the TE parameters at high temperatures from 300 to 525 K. There is a slight deviation for the obtained TE data near 300 K from these two methods probably due to the different gas and vacuum environments used. The electrical conductivity decreases monotonously with increasing temperature over the entire temperature range, showing a metal‐like behavior, as presented in **Figure** **1**a. The positive *S* indicates that the studied material is a p‐type semiconductor (Figure 1b). Additionally, the decrease in the *S* at higher temperature is a result of the intrinsic excitations. Figure 1c shows the temperature‐dependent κ. The κ increases first and then decreases with temperature because a grain boundary scattering dominates at *T* < 100 K followed by a three‐phonon scattering via the umklapp process.^[^ ^3^, ^30^, ^38^ ^]^ This typical behavior was also observed in other TE materials, for example, PbSe,^[^ ^31^ ^]^ Bi_2_Se_3_,^[^ ^39^ ^]^ and PdS.^[^ ^40^ ^]^ The κ increases with increasing temperature when the material is heated above 300 K, which is mainly related to the intrinsic excitations at high temperatures. As a result, a maximum power factor of  3.1 mW m^−1^ K^−2^ and a *zT* value of ≈1.0 were obtained at 300 K for Bi_0.5_Sb_1.5_Te_3_ (Figure 1d).

**Figure 2:**
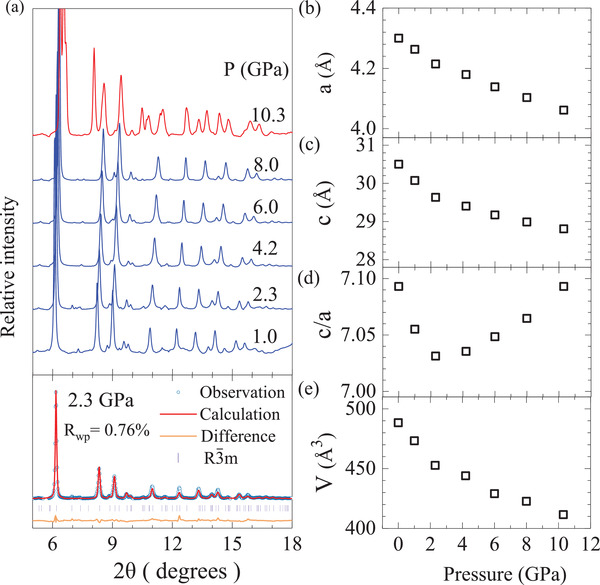
High‐pressure structural properties of Bi_0.5_Sb_1.5_Te_3_ at room temperature. a) X‐ray diffraction patterns at various pressures up to 10.3 GPa. The lower panel shows the Rietveld refinement of XRD pattern at pressure of 2.3 GPa. Pressure‐dependent lattice parameter b) *a*, c) *c*, d) axial ratio *c*/*a*, and e) volume.

**Figure 3:**
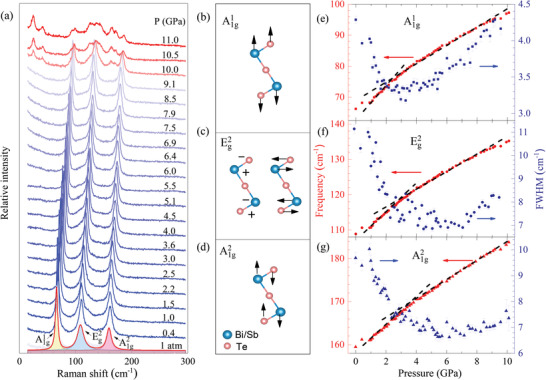
High‐pressure vibrational properties of Bi_0.5_Sb_1.5_Te_3_ at room temperature. a) Raman scattering spectra at various pressure up to 11.0 GPa. The vibration directions of the atoms are drawn for b) $A_{1g}^{1}$, c) $E_{g}^{2}$, and d) $A_{1g}^{2}$. Pressure‐dependent frequency (red, left panel) and FWHM (blue, right panel) of the phonon mode for e) $A_{1g}^{1}$, f) $E_{g}^{2}$, g) and $A_{1g}^{2}$. The dashed line is the fitting to the experimental data to give the pressure coefficient of each mode.

Since the crystal and electronic structure of the materials are easy to be modified by the applied pressure, we investigated the structural behavior of Bi_0.5_Sb_1.5_Te_3_ at different pressures. **Figure** **2**a presents the room‐temperature X‐ray diffraction (XRD) of Bi_0.5_Sb_1.5_Te_3_ at different pressures (see the XRD at 1 atm in Extended Data Figure S3, Supporting Information) and the Rietveld refinements of the collected XRD patterns at 2.3 GPa with a lattice parameter *a* = 4.2106 (0.0006) Å and *c* = 29.6032 (0.0125) Å. At ambient condition, the Bi_0.5_Sb_1.5_Te_3_ alloy crystallizes in a rhombohedral structure with space group *R* $\bar{3}$m, and this initial structure can be maintained up to 10.3 GPa and then enters the C2/m phase,^[^ ^41^, ^42^ ^]^ which is consistent with the observed Raman spectra (**Figure** **3**a). However, the C2/m phase is metallic and supposed to exhibit unsatisfactory TE performance. Therefore, we focus on the study of TE performance of the first phase. Based on the Rietveld refinement of XRD patterns, we calculated the lattice parameters and volume as a function of pressure, as shown in Figure 2b– e. The lattice parameters along the *a* and *c* axes gradually decrease with increasing pressure, indicating the shrinkage of the lattice and resulting in the reduced volume (Figure 2e). However, the *c*/*a* ratio decreases first and then increases with increasing pressure, reaching a minimum near 1.8 GPa. Meanwhile, the volume of the unit cell exhibits no discontinuity, as shown in Figure 2e. This abnormal behavior was also reported in Bi_2_Te_3_,^[^ ^43^ ^]^ Bi_2_Se_3_,^[^ ^44^ ^]^ and Sb_2_Te_3_,^[^ ^45^, ^46^ ^]^ and resulted from a change in the compressibility due to the existence of the ETT or Lifshitz transition.^[^ ^33^, ^45^ ^]^

The pressure‐dependent Raman spectra are shown in Figure 3a. The obvious phase transition occurs at pressure around 10 GPa, consistent with the XRD results. Three Raman‐active zone‐center phonon modes including one transverse (*E*_*g*_) and two longitudinal (*A*_1*g*_) modes are observed. Figure 3b– d show the vibration mode of three Raman‐active modes. The lowest‐frequency mode $E_{g}^{1}$, which also belongs to the *R* $\bar{3}$m phase is absent, as indicated in the previous Raman scattering measurements.^[^ ^41^, ^47^ ^]^ Figure 3e,f show respectively the frequency (left panel) and full width at half maximum (right panel) of the Raman mode of $A_{1g}^{1}$, $E_{g}^{2}$, and $A_{1g}^{2}$ by Lorentz fitting. All of the peaks harden with increasing pressure. The different pressure coefficient (the slopes of pressure‐dependent frequency curves, see dashed lines in Figure 3e,f) of three modes below and above 1.8 GPa is probably due to the existence of ETT, which has been proved in Bi_2_Te_3_‐based alloys.^[^ ^41^, ^46^, ^48^ ^]^ Furthermore, the pressure‐dependent full width at half maximum of the three modes also exhibits anomalous behaviors around 1.8 GPa.^[^ ^41^, ^45^ ^]^ Therefore, our Raman finding together with XRD results gives support to the observation of the ETT around 1.8 GPa in Bi_0.5_Sb_1.5_Te_3_.

**Figure 4:**
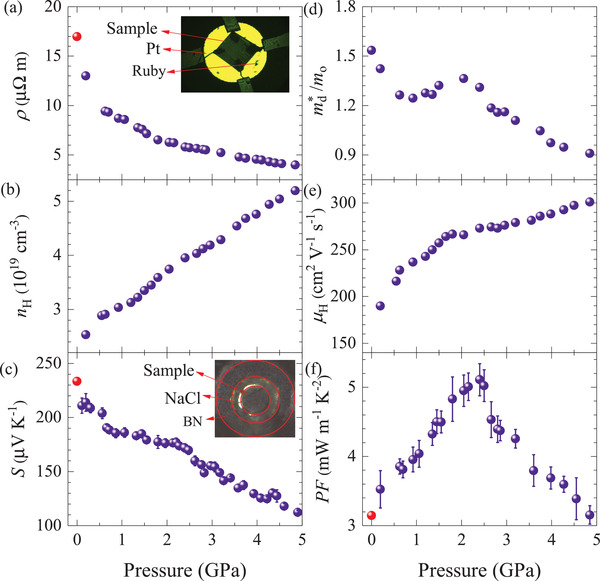
Pressure‐dependent a) electrical resistivity (ρ), b) Hall carrier concentration ($n_{H}$), c) Seebeck coefficient (*S*), d) density of states effective mass ($m_{d}^{*}$/*m*_0_), e) Hall mobility (μ_H_), and f) PF of Bi_0.5_Sb_1.5_Te_3_. The red dot is measured by CTA at ambient pressure. The inset in (a) shows the electrical transport measurements with daphne oil 7373 as pressure transmitting medium, and the inset in (c) displays the Seebeck coefficient measurements with NaCl as pressure transmitting medium. The error bars correspond to the uncertainties of the measured data.

**Figure** **4** shows the pressure‐dependent electrical properties of Bi_0.5_Sb_1.5_Te_3_. As shown in Figure 4a, the room‐temperature electrical resistivity decreases sharply with increasing pressure until ≈1.8 GPa, and then decreases slowly. Since there is no structural transition ≈1.8 GPa (Figure 2a), the abnormal trend ≈1.8 GPa is probably related to the ETT, which has also been confirmed by first‐principles calculations.^[^ ^36^, ^49^ ^]^ The ambient‐pressure ρ of Bi_0.5_Sb_1.5_Te_3_ is  17 μΩm, and it decreases to  3.9 μΩm at 5 GPa probably due to the decreased band gap with increasing pressure.^[^ ^30^, ^50^ ^]^ The decreased band gap leads to the increase in the Hall carrier concentration (*n*_H_), as can be seen in Figure 4b. The *n*_H_ increases from  2.53 × 10^19^ cm^−3^ at 0.2 GPa to  5.20 × 10^19^ cm^−3^ at 4.85 GPa. Figure 4c presents the pressure‐dependent *S* of Bi_0.5_Sb_1.5_Te_3_. The *S* in the whole pressure range is positive, indicating p‐type conduction. With increasing pressure, the *S* gradually decreases from  233.5 μV K^−1^ at 0 GPa to  112 μV K^−1^ at 5 GPa with a platform around 1.8 GPa. According to the equation

(1) $$S=\frac{8\pi^{2}k_{B}^{2}}{3eh^{2}}m_{d}^{*}{\left(\frac{\pi }{3n}\right)}^{2/3}$$

where *k*_B_ is the Boltzmann constant, *e* is the carrier charge, *n* is the carrier concentration, *h* is the Planck constant, and $m_{d}^{*}$ is the density of states effective mass. The larger $m_{d}^{*}$ leads to the larger *S*. The $m_{d}^{*}$ was calculated based on the single parabolic band model (The calculation details are given in the Note S3, Supporting Information) and presented in Figure 4d. The $m_{d}^{*}$ increases with increasing pressure at 1–2 GPa, which contributes to the enhanced *S* in spite of the increased *n*_H_ (Figure 4b). The change of the *S* with external pressure is the typical feature expected of the ETT.^[^ ^32^, ^50^ ^]^ Since the *S* are proportional to the derivative of the density of states at the Fermi level, it is strongly affected by the ETT. The ETT is beneficial to improve both the *S* and σ because it can increase the number of valleys in the Brillouin zone.^[^ ^32^, ^51^ ^]^ With increasing pressure, the Hall mobility increases from  190 to  301 cm^2^ V^−1^ s^−1^ (Figure 4e). Figure 4f shows the pressure‐dependent power factor of Bi_0.5_Sb_1.5_Te_3_. The ambient‐pressure power factor is  3.1 mW m^−1^ K^−2^, and it increases to  5.1 mW m^−1^ K^−2^ at around 2.4 GPa and exhibits a λ behavior.

**Figure 5:**
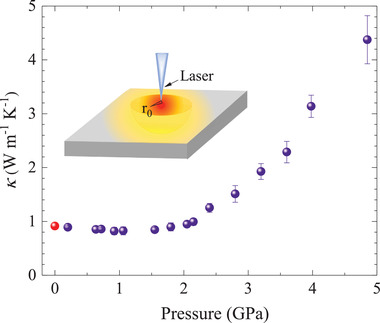
High‐pressure thermal conductivity of Bi_0.5_Sb_1.5_Te_3_ at room temperature. (Insert: the schematic diagram for thermal conductivity measurement) The red dots indicate the data at ambient pressure measured by LFA. The error bars correspond to the uncertainties of the measured data.

**Figure** **5** presents the pressure‐dependent total κ of Bi_0.5_Sb_1.5_Te_3_. The high‐pressure κ was measured using the Raman scattering method by changing the temperature and laser power (Extended Data Figure S4, Supporting Information). Because of the coupling of the related phonons and electrons, the frequency of Raman modes always varies with temperature. Therefore, the laser‐induced local temperature rise can be read indirectly by the Raman shift. Within the ideal heat diffusion model, the κ can be expressed as^[^ ^29^, ^31^, ^34^, ^52^, ^53^ ^]^

(2) $$\kappa =\eta \frac{W}{2\pi r_{0}\Delta T}$$

where *W* is the laser power, η is the absorption coefficient of the laser power, *r*_0_ is the width of the laser beam on the sample, ΔT is the temperature difference between the laser spot and the sample edge. The temperature (or laser power) dependence of the phonon modes can be written as χ_*T*_=(ω_*T*_‐ω_0_)/ΔT and χ_*w*_=(ω_*w*_‐ω_0_)/Δw, where χ_*i*_ is the first‐order temperature (or laser power) coefficient, Δw is the phonon frequency shift due to the variation of temperature or laser power. Thus, κ can be written as

(3) $$\kappa =\eta \frac{\chi_{T}}{2\pi r_{0}\chi_{w}}$$

Here, η is assumed to be insensitive to pressure and can be estimated from the comparison of the value of the ambient pressure determined by an independent technique such as laser flash technique. This assumption will not affect the studied trend with pressure. Our results display that the κ slightly declines with increasing pressure at 0–1.8 GPa, and then increases sharply upon further compression. A low κ is obtained around 0.89 W m^−1^ K^−1^ at 1.8 GPa. This slight reduction may be related to the enhanced effective phonon scattering by the lattice distortion in the vicinity of the ETT.

**Figure 6:**
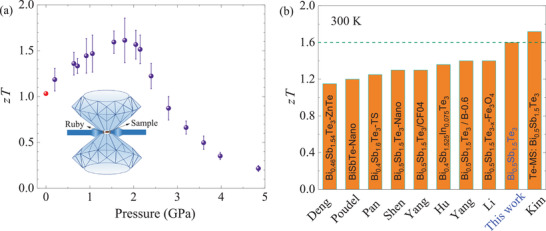
a) Pressure‐dependent *zT* of Bi_0.5_Sb_1.5_Te_3_ at 300 K. The red dot represents the calculated value using the data measured by CTA and LFA at ambient pressure. Inset: the vehicle for measuring high‐pressure thermoelectric properties. The error bars correspond to the combined uncertainties of the power factor and total thermal conductivity. b) The comparison of our obtained peak *zT* at room temperature with the other high‐*zT* p‐type Bi_0.5_Sb_1.5_Te_3_ materials.^[^ ^8^, ^20^ ^]^^[^ ^22^, ^25^, ^26^, ^27^ ^]^^[^ ^54^, ^55^, ^56^ ^]^

The pressure dependence of *zT* of Bi_0.5_Sb_1.5_Te_3_ at room temperature is shown in **Figure** **6**a. Benefiting from the enhanced power factor due to the existence of ETT and slightly reduced κ at ≈1.8 GPa, the *zT* is significantly enhanced from  1.0 at ambient pressure to  1.6 at 1.8 GPa. An increase of ≈60 % for *zT* at room temperature is realized in this p‐type model thermoelectric material just through lattice contraction. To better highlight the advantages, we have compared our result with the representative p‐type Bi_0.5_Sb_1.5_Te_3_ materials are shown in Figure 6b.^[^ ^8^, ^20^, ^22^, ^25^, ^26^, ^27^, ^54^, ^55^, ^56^ ^]^ Clearly, the *zT* value of our Bi_0.5_Sb_1.5_Te_3_ material at 300 K outperforms almost all the best reported materials and is comparable even to the best reported by Kim et al.^[^ ^20^ ^]^ The pressure‐driven ETT turns out to be an effective way to tune the electronic structure for the improved TE properties while maintaining the same crystal structure.

## 3 Conclusion

In summary, thermoelectric properties of p‐type Bi_0.5_Sb_1.5_Te_3_ alloy have been investigated at high pressures. By tuning the external pressure, we have obtained a room‐temperature record high *zT* of 1.6 at the pressure of 1.8 GPa. The pressure‐driven electronic topological transition is proposed to account for such an enhancement. High‐pressure in situ measurements of X‐ray diffraction, Raman scattering, electrical transport properties at different pressures support the occurrence of electronic topological transition. The present results demonstrate that driving the compounds to enter the electronic topological transition conditions is a new guide for further improving the thermoelectric properties.

## 4 Experimental Section

### Sample Preparation

The Bi_0.5_Sb_1.5_Te_3_ sample was prepared by melting, ball milling, and hot pressing. Elements of bismuth (Bi, 99.999%, shots), antimony (Sb, 99.999%, shots), and tellurium (Te, 99.999%, shots) were weighed according to the nominal composition, sealed into a quartz tube at 10^−5^ Pa, and then melted for 10 h in a box furnace at 1023 K. The obtained ingot was cleaned and ground into powder by ball milling (SPEX 8000M) for 4 h. The obtained powder was loaded into a graphite die with an inner diameter of 12.7 mm and consolidated at 723 K for 2 min by a direct current‐induced hot pressing under an axial pressure of 50 MPa.

### Structural Characterization

The ambient‐pressure crystal structure was determined by XRD on a Rigaku diffractometer (Rigaku D/max 2500 PC) with a Cu Kα radiation source. For the high‐pressure structural study, synchrotron XRD patterns were collected at the GeoSoilEnviroCARS (Advanced Photon Source, Argonne National Laboratory, USA) with the wavelength of 0.3100 Å. Neon was loaded together with the sample to serve as the pressure transmitting medium. The Fit2D software^[^ ^57^ ^]^ was used to convert the obtained 2D XRD data to the 1D profile. These XRD data were further analyzed through the Rietveld refinement with the GSAS program package.^[^ ^58^ ^]^ The microstructures were investigated by a scanning electron microscope (Phenom pro). The grain size was decreased to less than 1 μm by ball milling to weaken the anisotropy (Extended Data Figure S1, Supporting Information).

### Ambient‐Pressure Thermoelectric Properties Characterization

The electrical resistivity (ρ) and *S* at 300–525 K were simultaneously measured on a commercial system (CTA‐3). The κ was calculated by κ = Dα *C*_p_, where D is the volumetric density determined by the Archimedes method, α is the thermal diffusivity obtained by a laser flash apparatus (Netzsch; LFA 457), and *C*_p_ is the specific heat taken from the previous report.^[^ ^59^ ^]^ The ρ, *S*, and κ at 2–300 K were measured in a thermal transport option (TTO) setup using a PPMS by Quantum Design.

### High‐Pressure Thermoelectric Properties Characterization

The sample was mounted in a nonmagnetic diamond anvil cell made of Cu–Be alloy at various interesting pressures. The ρ and Hall effect were measured on a PPMS based on the standard four‐probe technique. Daphne oil 7373 was loaded into the sample chamber to provide the pressure transmitting medium. The van der Pauw method^[^ ^60^ ^]^ was used to determine the resistivity, conductivity, and *n*_H_. The high‐pressure *S* was measured based on the definition *S* = −ΔV/ΔT (Extended Data Figure S2, Supporting Information) with ΔV being of thermoelectric voltage and ΔT being of temperature gradient. The K‐type thermocouple was selected to measure the temperature gradient between the hot side and the cold side. Meanwhile, the thermoelectric voltage was collected by a digital nanovoltmeter (218‐A‐5900, Keithley). The κ was measured using the Raman scattering method by changing the temperature and laser power.

### High‐Pressure Raman Scattering Measurements

Raman spectra were measured using a single‐stage spectrograph equipped with an array thermoelectrically cooled charge‐coupled device (CCD) detector. The 488 nm excitation was used on the sample. The scattered light was dispersed by an 1800 groves mm^−1^ grating and collected by a CCD resulting in a spectral resolution of  1 cm^−1^. In order to avoid the potential over‐heating or oxidation of the samples, the highest laser power of 2.8 mW was set for the measurements. Neon was again used as the transmitting medium to create the same pressure environments for comparisons. In all the above high‐pressure experiments, the fluorescence spectra of the ruby were collected near room temperature to calibrate the pressure level.^[^ ^61^ ^]^

## Conflict of Interest

The authors declare no conflict of interest.

## Supporting information

Supporting Information

## Data Availability

the data that support the finding of this study are available from the corresponding author upon reasonable request.
