# Supplementary material for: Electronic Topological Transition as a Route to Improve Thermoelectric Performance in Bi0.5Sb1.5Te3
Source: Adv Sci (Weinh). 2022 Mar 15;9(14):2105709. doi: 10.1002/advs.202105709 (PMC9108658; doi:10.1002/advs.202105709)
Supplement: Supplementary file 1 — Supporting Information [file ADVS-9-2105709-s001.pdf]

ADVANCED SCIENCE
Open Access

Supporting Information

for *Adv. Sci.*, DOI 10.1002/advs.202105709

Electronic Topological Transition as a Route to Improve Thermoelectric Performance in $Bi_{0.5}Sb_{1.5}Te_3$

Feng-Xian Bai, Hao Yu, Ya-Kang Peng, Shan Li, Li Yin, Ge Huang, Liu-Cheng Chen, Alexander F. Goncharov, Jie-He Sui, Feng Cao, Jun Mao, Qian Zhang* and Xiao-Jia Chen**

ADVANCED SCIENCE
Open Access

Supporting Information

for *Adv. Sci.*, DOI: 10.1002/advs.202105709

Electronic Topological Transition as a Route to the Improvement of Thermoelectric Performance in $Bi_{0.5}Sb_{1.5}Te_3$

Feng-Xian Bai[1,2], Hao Yu[2,3], Ya-Kang Peng[2], Shan Li[1], Li Yin[1], Ge Huang[2], Liu-Cheng Chen[2,3], Alexander F. Goncharov[4], Jie-He Sui[5], Feng Cao[3], Jun Mao[1,5*], Qian Zhang[1,5*], Xiao-Jia Chen[2,3*]

Supplementary Information

Electronic Topological Transition as a Route to the Improvement of Thermoelectric Performance in $Bi_{0.5}Sb_{1.5}Te_3$

Feng-Xian Bai[1,2], Hao Yu[2,3], Ya-Kang Peng[2], Shan Li[1], Li Yin[1], Ge Huang[2], Liu-Cheng Chen[2,3], Alexander F. Goncharov[4], Jie-He Sui[5], Feng Cao[3], Jun Mao[1,5*], Qian Zhang[1,5*], Xiao-Jia Chen[2,3*]

[1]*School of Materials Science and Engineering, and Institute of Materials Genome & Big Data, Harbin Institute of Technology, Shenzhen 518055, China*

[2]*Center for High Pressure Science and Technology Advanced Research, Shanghai 201203, China*

[3]*School of Science, Harbin Institute of Technology, Shenzhen 518055, China*

[4] *Earth and Planets Laboratory, Carnegie Institution for Science, Washington, DC 20015, USA*

[5]*State Key Laboratory of Advanced Welding and Joining, Harbin Institute of Technology, Harbin 150001, China*

E-mail: maojun@hit.edu.cn, zhangqf@hit.edu.cn, xjchen@hit.edu.cn and xjchen@hpstar.ac.cn

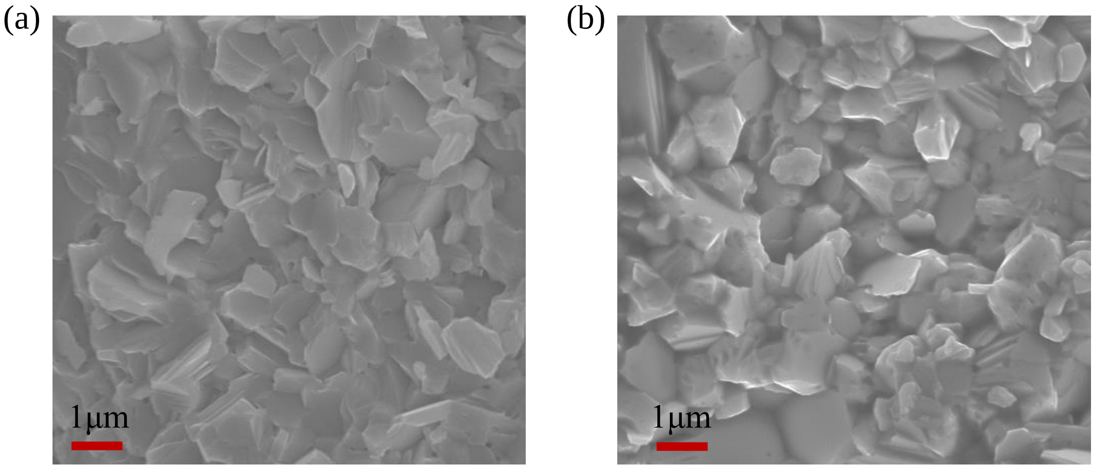

Figure S1. SEM images of $Bi_{0.5}Sb_{1.5}Te_3$ at (a) ambient pressure, (b) the pressure release.

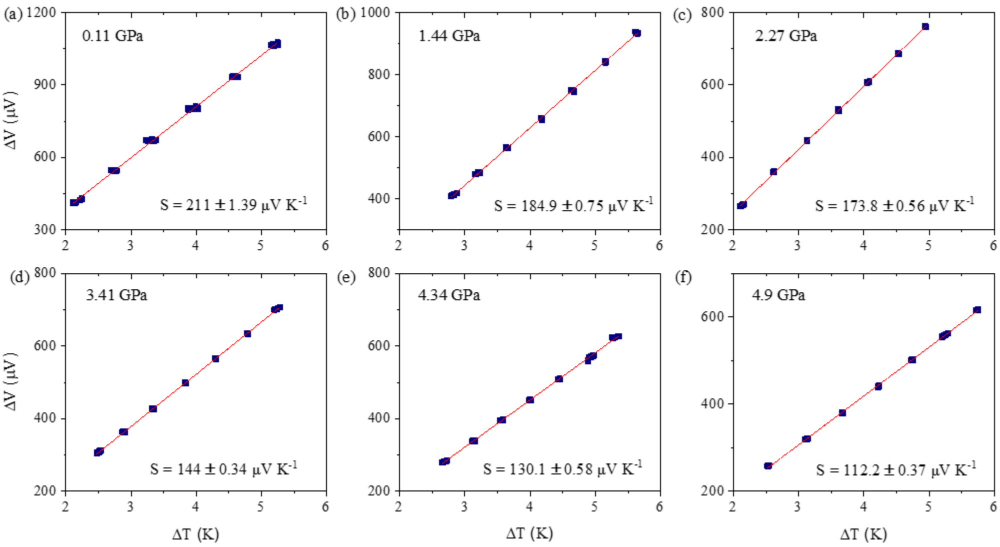

Figure S2. The voltage difference Δ*V* vs temperature gradient Δ*T* relations of $Bi_{0.5}Sb_{1.5}Te_3$ at (a) 0.11GPa, (b) 1.44GPa, (c) 2.27GPa, (d) 3.41GPa, (e) 4.34GPa, (f) 4.9 GPa at room temperature. Linear fitting to the data points gives the *S* value for each measured pressure.

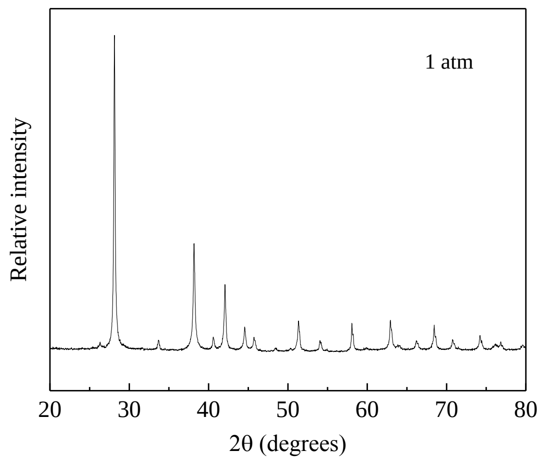

Figure S3. X-ray diffraction patterns of $Bi_{0.5}Sb_{1.5}Te_3$ at 1atm.

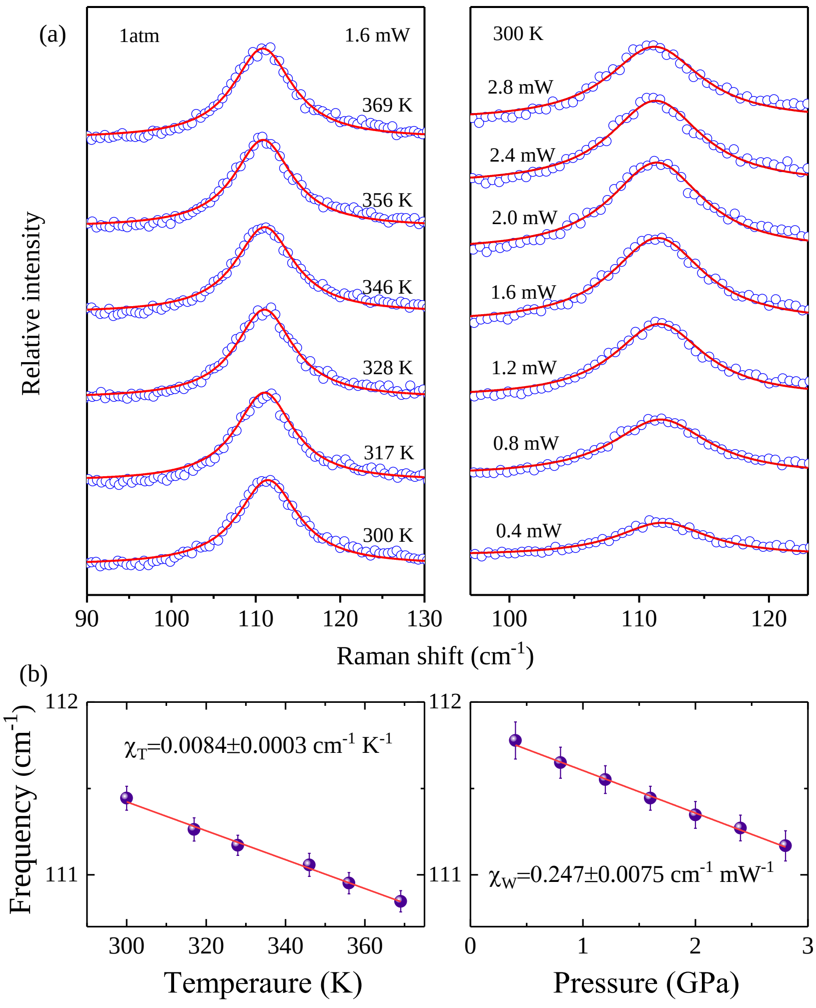

Figure S4. High-pressure thermal conductivity measurements of $Bi_{0.5}Sb_{1.5}Te_3$ by Raman. (a) Raman spectra of the E_g^2 phonon mode at different temperature (left panel) and different laser power (right panel) measured at 1 atm. (b) Raman frequency of the E_g^2 phonon mode as functions of temperature and laser power at 1 atm. χ_T and χ_W were obtained from the linear fitting to the data. The error bars correspond to the uncertainties of the measured data. The error bars correspond to the uncertainties of the measured data.

Supplementary Note 1: High-pressure electrical resistivity and Hall coefficient measurements. The high-pressure electrical resistivity and Hall coefficient were measured by PPMS based on the standard four-probe technique in a nonmagnetic diamond anvil cell (DAC) made of Cu-Be alloy. This customized cell has a pair of diamond anvils and the diameter of the anvil culets is 500 µm. A steel flake was used as the gasket. The gasket was drilled a hole with a diameter of 500 µm in the centre of pre-indented area. The gasket was insulated with Cubic BN (c-BN) fine powders that was pressed into pre-indented area and the drilled hole. Consequently, another hole of 300 mm in diameter was drilled at the centre of the 500 mm hole filled with c-BN, which was then further filled with silicone oil as the pressure-transmitting medium. A piece of the polycrystalline sample was placed into the sample chamber. Four Pt wires were placed on the surface of one side of the insulated gasket with the linkage of the sample and the external Cu wires. The resistivity and Hall coefficient were determined in terms of the van der Pauw method [1]. For this method, the thickness of the measured sample is the only needed dimensional parameter. The sample thickness for the electrical transport measurements is 33 µm. No pressure transmitting medium was used for such electrical transport measurements. The loaded ruby chip was used to determine pressure. The Hall effect measurements were taken at room temperature.

Supplementary Note 2: High-pressure Seebeck coefficient measurements. The high-pressure Seebeck coefficient was measured based on the definition S = -$\Delta V/\Delta T$. For applying external heating, the K-type thermocouple is selected to measure the temperature gradient between the hot side and the cold side. Meanwhile, the thermoelectric voltage is collected by a digital nanovoltmeter (218-A-5900,

Keithley). The sample is loaded in the hole of gasket that was insulated with Cubic BN (c-BN) fine powders, which was then further filled with NaCl as the pressure-transmitting medium.

Supplementary Note 3: Single parabolic band (SPB) model: The calculation details of the density-of-states effective mass based on the single parabolic band (SPB) model [2,3] are shown as follows:

$$S=\pm\frac{k_B}{e}\left[\frac{(r+5/2)F_{r+3/2}(\eta_F)}{(r+3/2)F_{r+1/2}(\eta_F)}-\eta_F\right] \quad (1)$$

$$F_i(\eta_F)=\int_0^{\infty}\frac{x^i dx}{1+exp(x-\eta_F)} \quad (2)$$

$$\eta_F=E_F/k_BT \quad (3)$$

$$r_H=\frac{3}{2}F_{1/2}(\eta_F)\frac{(1/2+2\lambda)F_{2\lambda-1/2}(\eta_F)}{(1+\lambda)^2F_{\lambda}^2(\eta_F)} \quad (4)$$

$$m_d^*=\frac{h^2}{2k_BT}\left(\frac{n_Hr_H}{4\pi F_{1/2}(\eta_F)}\right)^{2/3} \quad (5)$$

$$L=\left(\frac{k_B}{e}\right)^2\left\{\frac{(r+7/2)F_{r+5/2}(\eta_F)}{(r+3/2)F_{r+1/2}(\eta_F)}-\left[\frac{(r+5/2)F_{r+3}(\eta_F)}{(r+3/2)F_{r+1/2}(\eta_F)}\right]^2\right\} \quad (6)$$

where k_B is the Boltzmann constant, e is the electron charge, η_F is the reduced Fermi energy, r is the scattering factor (here, acoustic phonon scattering is considered, r = -1/2, $F_i(\eta_F)$ is the Fermi-Dirac integral, r_H is the Hall factor, λ is related to the scattering parameter r, such that $\lambda=r+1/2$, m_d^* is the density-of-states (DOS) effective mass. According to the experimental Seebeck coefficient values, the reduced Fermi level η_F can be deduced using equation (1) and equation (2). Substituting the estimated η_F and the measured Hall carrier concentration into equation (4) and equation (5), the effective mass can be obtained. We also calculated the Lorenz number based on the SPB model using equation (6).

References

[1] L. J. V. d. Pauw, Philips Res. Rep. **13**, 1 (1958).

[2] J. Mao, H. S. Kim, J. Shuai, Z. Liu, R. He, U. Saparamadu, F. Tian, W. Liu, and Z. Ren, Acta

Mater. **103**, 633 (2016).
[3] W. Li, B. Zhou, J. Li, S. Zhu, and J. Li, J. Alloys Compd. **753**, 93 (2018).
